# Intravenous immunoglobulins for treatment of severe COVID-19-related acute encephalopathy

**DOI:** 10.1007/s00415-022-11152-5

**Published:** 2022-05-10

**Authors:** Shufan Huo, Caroline Ferse, Fabian Bösl, S. Momsen Reincke, Philipp Enghard, Carl Hinrichs, Sascha Treskatsch, Stefan Angermair, Kai-Uwe Eckardt, Heinrich J. Audebert, Christoph J. Ploner, Matthias Endres, Harald Prüss, Christiana Franke, Franziska Scheibe

**Affiliations:** 1 Department of Neurology and Experimental Neurology, Charité-Universitätsmedizin Berlin, Freie Universität Berlin and Humboldt-Universität zu Berlin, Charitéplatz 1, 10117 Berlin, Germany; 2 Center for Stroke Research Berlin, Charité-Universitätsmedizin Berlin, Berlin, Germany; 3 DZHK (German Centre for Cardiovascular Research), partner site Berlin, Berlin, Germany; 4 Department of Nephrology and Medical Intensive Care, Charité-Universitätsmedizin Berlin, Freie Universität Berlin and Humboldt-Universität zu Berlin, Berlin, Germany; 5 German Center for Neurodegenerative Diseases (DZNE), Berlin, Germany; 6 Department of Anesthesiology and Intensive Care Medicine, Charité-Universitätsmedizin Berlin, Freie Universität and Humboldt-Universität zu Berlin, Berlin, Germany; 7 Berlin Institute of Health at Charité-Universitätsmedizin Berlin, Berlin, Germany; 8 NeuroCure Cluster of Excellence, Charité-Universitätsmedizin Berlin, Freie Universität Berlin and Humboldt Universität zu Berlin, Berlin, Germany

Dear Sirs,

Coronavirus disease 2019 (COVID-19) patients on intensive care units (ICU) frequently present with acute encephalopathy that appears to be distinct from other ICU-related encephalopathies regarding higher incidence, longer duration, and increased severity including autonomous dysregulation and non-satisfactory response to neuroleptic drugs [1]. According to the updated nomenclature of delirium and acute encephalopathy, we use the term acute encephalopathy to describe a rapidly developing pathobiological brain process, expressed clinically as delirium, subsyndromal delirium or coma with partly additional neurological findings such as extrapyramidal signs or seizures [2]. This type of acute encephalopathy is associated with delayed recovery, weaning failure, prolonged ICU or hospital stay, or even impaired clinical outcome.

First reports found positive responses of COVID-19-associated encephalopathy to immunotherapy [3–5] that supported the hypothesis of a possible inflammatory pathomechanism [6]. However, this encephalopathy might still be misjudged by intensivists as poorly treatable with limited prognosis, risking premature withdrawal of ICU therapy or even end-of-life decisions in affected patients. Therefore, our case series demonstrates a promising and rapid effect of intravenous immunoglobulins (IVIg) on otherwise treatment-refractory acute encephalopathy in COVID-19 patients on ICU.

This retrospective, single-center case series included 12 patients with critical courses of COVID-19 requiring treatment at ICU, who developed a severe encephalopathy (leading to clinical presentation of hyper- and/or hypoactive delirium [2]) of at least 1 week without satisfactory response to neuroleptic drugs and/or even sedatives. These patients were treated with 2 g/kg IVIg over 3–5 days as off-label, individual medical treatment. The dosage of IVIg was chosen pragmatically according to established therapeutic regimens in other neurological autoimmune-mediated diseases.

Other causes of encephalopathy such as increased blood levels of sedative medication, intoxication, metabolic changes (abnormal electrolyte concentrations, hyperuricemia, hepatic encephalopathy, hypo- or hyperglycemia), sepsis with fever, hypothermia, shock or hypoxia were excluded. All patients received treatment with 6 mg dexamethasone for at least 10 days according to the RECOVERY study protocol [7].

After occurrence of acute encephalopathy and prior to IVIg therapy, all patients received imaging with either cerebral CT or MRI and one-time cerebrospinal fluid (CSF) examinations (except patient #8, whose critical clinical status did not permit lumbar puncture) with measurement of standard laboratory parameters. Antineuronal autoantibodies (namely: IgG-antibodies against amphiphysin, PNMA2 (Ma2/Ta), Ri, Yo, Hu, CV2 (CRMP5), Tr (DNER), NMDA receptor, GABA-b receptor, AMPA receptor1/2 (GluA1/GluA2), mGluR5, Glycin receptor, Dopamin2 receptor, DPPX, LGI1, CASPR2, Aquaporin-4, Myelin, GAD65) were determined by cell-based indirect immunofluorescence assays at Labor Berlin or Euroimmun, Germany. A PCR-screening for common neurotropic pathogens (namely: SARS-CoV-2, herpes simplex virus 1/2, varicella zoster virus, human herpes virus 6, Epstein–Barr virus, cytomegalovirus, enterovirus, parechovirus, *Streptococcus pneumoniae*, *Neisseria meningitidis*, *Haemophilus influenzae*, *Listeria monocytogenes*, group B streptococcus, *Escherichia coli* (K1), and cryptococcus) excluded other common CNS infections. Additionally, indirect immunofluorescence technique on unfixed murine brain sections was applied with CSF and serum according to previously published protocols [8]. The inflammatory marker IL-6 was measured routinely every other day with Elecsys^®^ IL-6 immunoassay (Roche) and Immulite IL-6 (Siemens). Neurofilament light chain was determined in serum with Simoa NF-light™ assay (Quanterix) and in CSF with NF-light™ ELISA (UmanDiagnostics).

Clinical neurological outcome was continuously evaluated by a neurologist and reported immediately before initiation and after termination of IVIg therapy and at discharge. Outcomes were assessed by Confusion Assessment Method for ICU (CAM-ICU), Richmond Agitation–Sedation Scale (RASS), Glasgow Coma Scale (GCS), Glasgow Outcome Scale (GOS) and modified Rankin Scale (mRS). Standardized scores were primarily ascertained by the treating neurologist. In case of missing data, scores were obtained from the daily routine assessment by the ICU nurses.

The patient cohort (*n* = 12) had a median age of 67 years (range 43–77, two females), required a median of 61 days (range 33–160) of ICU treatment and presented high rates of multi-organ failure with invasive ventilation in 11 (92%, *n* = 9 prone positioning, *n* = 2 veno-venous extracorporeal membrane oxygenation), pulmonary superinfections in 11 (92%), hepatic failure in five (42%), renal replacement therapy in nine (75%) and catecholamine therapy in all patients (Table 1). After a median of 23 days (range 0–37) after hospital admission due to COVID-19, all patients developed encephalopathy, requiring continuous intravenous sedation in 10 patients (83%, Table 2).

**Table 1:** Clinical presentation and duration of the disease

#	Age (y)	Sex	Clinical features	Past medical history	BMI on admission	Pulmonary superinfection	Invasive ventilation	Prone positioning	vvECMO	Hepatic failure	RRT	Sepsis	Catecholamine therapy	Hospital stay (d)	ICU stay (d)
1	77	m	Hyperactive delirium	aHT, CAD, obesity	34	+	+	+	–	–	+	+	+	81	81
2	75	m	Hyperactive delirium, seizures	aHT, AF, pulmonary embolism	25	–	–	–	–	–	–	–	+	38	37
3	62	m	Hypoactive delirium	aHT, DM2, smoking, obesity	30	+	+	+	–	–	+	+	+	76	73
4	73	m	Hypoactive delirium	myasthenia gravis	22	+	+	+	–	–	+	+	+	58	57
5	75	m	Hypoactive delirium	aHT, DM2, MI, aortic valve replacement	28	+	+	+	–	–	-	+	+	54	50
6	74	m	Hyperactive delirium, seizures, myoclonia	aHT, AF, renal transplant, obesity	32	+	+	–	–	+	+	+	+	195	64
7	52	f	Hypoactive delirium	aHT	27	+	+	–	–	–	+	+	+	55	55
8	43	m	Hyperactive delirium	obesity	31	+	+	+	+	–	–	+	+	103	100
9	49	m	Hyperactive delirium	aHT, DM2, sarcoidosis, obesity	30	+	+	+	+	+	+	+	+	102	99
10	59	f	Hypoactive delirium, myoclonia	aHT, DM2, obesity	34	+	+	+	–	+	+	+	+	Unknown	160
11	55	m	Hypoactive delirium	aHT, renal failure with RRT	22	+	+	+	–	+	+	+	+	57	53
12	72	m	Hypoactive delirium	aHT, CAD, MI	26	+	+	+	–	+	+	+	+	Unknown	33
													Median	67	60.5

Clinical features (pulmonary superinfection, invasive ventilation, proning, vvECMO, hepatic failure, RRT, sepsis, catecholamine therapy) are listed as “+” if they occurred at any time during the ICU stay. The term “delirium” is used in this manuscript to describe the clinical presentation of acute encephalopathy[2]. Before IVIg treatment, other sources of encephalopathy such as sepsis or hepatic failure were excluded at the time of clinical presentation of delirium, see exclusion criteria. For patients #10 and #12, the total duration of hospital stay could not be determined due to missing information about previous treatments in other hospitals. While 11 patients had at least one known cardiovascular risk factor, patient #4, who presented with known myasthenia gravis, did not. Patient #9 additionally suffered from stage II pulmonary sarcoidosis. Overall, most patients were severely affected by multiple complications (e.g., sepsis in 11, need for invasive ventilation in 11, renal failure with need of replacement therapy in nine, vvECMO in two patients)

− no, +  yes, *AF* atrial fibrillation, *aHT* arterial hypertension, *BMI* body mass index, *CAD* coronary artery disease, *d* days, *DM2* diabetes mellitus type 2, *f* female, *ICU* intensive care unit, *m* male, *MI* myocardial infarction, *RRT* renal replacement therapy, *vvECMO* veno-venous extracorporeal membrane oxygenation, *y* years

**Table 2:** Detailed clinical and time course: changes of CNS medication

#	Clinical status			CNS medication			Time delirium onset after admission (d)	Time delirium onset—IVIg start (d)	Time IVIg start—improvement (d)
	Before IVIg = t1	After IVIg = t2	Discharge = t3	t1	t2	t3			
**1**	Agitation, myoclonia, no GF	GF, RTC	Adequate communication, intermittent agitation	Levomepromazine, Lorazepam, Sufentanil	↓	↓	15	37	1
**2**	Agitation, no GF, no communication	RTC, disoriented but communication possible	→	Morphine, Risperidone	→	→	0	32	4
**3**	Drowsiness, no GF, vegetative stress upon weaning	RTC	Adequate communication, RTC, agitation, panic	Dexmedetomidine, Lormetazepam, Morphine, Propofol, Quetiapine	↓	↓	36	12	3
**4**	Intermittent GF, no communication, tetraplegia	→	No delirium	Morphine	↓	none	24	10	6
**5**	Coma, tetraplegia	Awake, GF, no RTC, moves feet	N/A	Amantadine, Morphine	→	N/A	14	39	5
**6**	Somnolent, GF, disoriented, no RTC, dysarthria, myoclonia	Awake, disoriented but communication possible, dysarthria improved	→	Risperidone	→	none	22	28	4
**7**	No GF, no response to stimulus	GF, RTC	No delirium	Clonidine, Sufentanil	none	Melperon	10	26	6
**8**	Severe agitation, skew deviation	Squints eyes after stimulus	No delirium	Dexmedetomidine, Esketamine, Morphine, Phenobarbital, Propofol	↓	↓	nd	nd	6
**9**	Agitation, no GF, myoclonia, brachiofacial dyskinesia	Awake, intermittent GF, RTC	GF, RTC, communication possible	Diazepame, Morphine, Risperidone	↓	↓	nd	nd	4
**10**	GF, RTC, visual hallucinations	→	N/A	Citalopram, Clonidine, Sufentanil	↑	N/A	30	24	N/A
**11**	No response to stimulus, tetraplegia	No RTC, moves arms and legs	N/A	Dexmedetomidine, Lorazepam, Quetiapin, Sufentanil	↑	N/A	23	15	N/A
**12**	Coma, eye deviation	→	N/A	Sufentanil	→	N/A	37	15	N/A
						**Median**	22.5	25	4

This table shows the detailed clinical course upon neurological examination as well as changes in application of CNS medication. N/A is stated if the patient died before discharge. Patients #2 and #6 were discharged immediately after IVIg treatment due to clinical improvement, which is why their clinical statuses at t2 and t3 remained unchanged. For patients #8 and #9 the exact time of acute encephalopathy diagnosis could not be determined due to continuous need for sedation

Clinical presentation of myoclonia: Patient #1 and #9: generalized, patient #6: fine myoclonia of the legs

↑ increased, ↓ decreased,  →  no change, *d* days, *GF* gaze fixation, *RTC* response to commands, *t1* last value before IVIg therapy, *t2* first value after IVIg therapy, *t3* last value before discharge or death

Cerebral CT or MRI showed none or only unspecific findings, but no abnormalities explaining the observed encephalopathy (Table 3). CSF was taken in 11 patients. In accordance with a recently published study about CSF findings in COVID-19 [9], eight of our patients showed blood brain barrier (BBB) dysfunction and all yielded normal cell counts and negative PCR for common pathogens including SARS-CoV-2. Furthermore, nine patients displayed either low-titer antineuronal antibodies in serum (Myelin, CASPR2, NMDAR, Yo), or IgG binding of unknown specificity in indirect immunofluorescence of blood and CSF on unfixed murine brain sections, or both (Table 3). Increased neurofilament light-chain values in serum and CSF of all investigated patients (measured in *n* = 11 of 12) reflected relevant neuronal degeneration or damage.

**Table 3:** Diagnostic findings

#	CT/MRI	EEG	CSF cells /µl	Oligoclonal bands CSF/serum	Reibergram	CSF glucose mg/dl	CSF lactate mg/dl	CSF total protein mg/l	Qalb	IgG %	IgA%	IgM%	
1	MRI: thickened meninges, white matter lesions	Alpha, intermittent theta rhythm	2	Type 4	Normal	85	16.7	294.6	6.9	0	0	0	
2	CT: chronic lacunar infarctions, massive leukoaraiosis	Alpha, intermittent theta rhythm	2	Type 1	Barrier dysfunction	55	16.7	792.1	14.4	0	0	0	
3	CT: mild leukoaraiosis	Theta, intermittent delta rhythm	1	Type 4	Normal	127	17.3	217.8	4.8	0	0	0	
4	MRI: small SAH, several microbleeds, small chronic infarction	Mixed theta-delta rhythm	7	Type 1	Barrier dysfunction	82	13.2	717.4	13.8	0	13	45	
5	MRI: two small acute infarctions	Theta, intermittent delta rhythm	1	Type 4	Borderline barrier dysfunction	136	25.1	354.6	9.3	0	0	0	
6	CT: leukoaraiosis	Theta, intermittent delta rhythm	2	Type 3	Barrier dysfunction	127	17.8	678.9	11.7	0	0	0	
7	CT: mild leukoaraiosis	Mixed theta-delta rhythm	0	Type 4	Barrier dysfunction	59	16.8	379	8.4	0	0	0	
8	CT: normal	nd	nd	nd	nd	nd	nd	nd	nd	nd	nd	nd	
9	CT: partially empty sella	nd	0	Type 1	Barrier dysfunction	97	17	864	21.5	0	0	0	
10	MRI: normal PET-CT: limbic encephalitis	nd	nd	Type 1	Barrier dysfunction	nd	nd	nd	26.8	0	35	51	
11	CT: leukoaraiosis	nd	2	Type 4	normal	70	17	239	7.7	0	0	0	
12	MRI: multiple chronic small infarctions, global atrophy, leukoaraiosis	Mixed theta–delta rhythm	1	Type 4	Borderline barrier dysfunction	101	16.1	556	25.8	0	nd	0	

#	Antineuronal antibodies serum	Antineuronal antibodies CSF	IIFT binding pattern serum	IIFT binding pattern CSF	NfL serum (pg/ml)	NfL CSF (pg/ml)	NfL Ratio	IL-6 before IVIg (ng/l)	IL-6 after IVIg (ng/l)	IL-6 at discharge/death (ng/l)
1	Myelin Ab 1:100	None	Panneuronal	Panneuronal	435	2112	4	56	59	83
2	nd	None	Negative	Negative	31	6359	205	252	26	27
3	Myelin Ab 1:100	None	Medium sized vessels, myelin	Medium sized vessels, Myelin	616	1181	1	19	23	7
4	None	None	Negative	negative	256	2445	9	24	30	nd
5	CASPR2&NMDAR IgG Ab 1:10	None	Antinuclear	Antinuclear	914	1683	1	105	116	99
6	None	None	Antinuclear, perinuclear	Negative	125	2264	18	37	nd	16
7	nd	None	Antinuclear, Perinuclear	Antinuclear, perinuclear	185	981	5	66	16	39
8	None	nd	Panneuronal	nd	733	nd	nd	238	107	13
9	nd	None	Negative	Negative	232	2223	9	89	40	24
10	Yo Ab	Yo Ab	nd	Antinuclear	nd	2215	nd	1087	nd	61
11	Myelin Ab 1:100	None	Negative	Negative	4153	12,500	3	121	133	5905
12	Myelin Ab 1:100, Glycin-R Ab 1:10	None	Negative	Negative	1348	28,099	20	106	237	-
			Mean		821	5642	28	183	79	627
			Responders		392	2406	32	98	52	39
			Non-responders		2751	14,271	12	438	185	2983
			*P* values		0.0023	0.0216	0.711	0.0819	0.0056	0.0342

All findings were measured after occurrence of acute encephalopathy and prior to IVIg administration except IL-6 levels, which were determined every second day throughout the ICU stay. Cerebral imaging only showed nonspecific pathologies including small ischemic strokes or intracerebral bleedings with no clinical correlation. Such imaging alterations are frequently seen in COVID-19 patients due to their higher risk of cerebral vasculopathy, especially in patients treated with vvECMO-therapy. But also activation of the coagulation system in COVID-19 patients predisposes them to thrombotic events of the brain and other organs. Patient #4 had a blood contamination in lumbar puncture with an increased CSF cell count of 7/µl and 1000 erythrocytes/µl. In all other patients CSF cell count and cytology were normal (< 5/µl, lymphocytes). Part of the CSF samples of patient #10 coagulated due to blood contamination, which is why some laboratory information is missing

Oligoclonal bands are reported as follows: type 1: Normal CSF, type 2: Oligoclonal IgG restricted to CSF, type 3: Oligoclonal IgG in CSF with additional identical bands in CSF and serum (combination of types 2 and 4), type 4: Identical oligoclonal bands in CSF and serum

Antineuronal antibodies in serum and CSF were tested according to a standard panel including IgG-antibodies against amphiphysin, PNMA2 (Ma2/Ta), Ri, Yo, Hu, CV2 (CRMP5), Tr (DNER), NMDAR, GABA-b-R, AMPA-R1/2 (GluA1/GluA2), mGluR5, Glycin-R, Dopamin2-R, DPPX, LGI1, CASPR2, Aquaporin-4, Myelin, GAD65. The upper limit for normal serum NfL levels is 9.9 pg/ml. The values for CSF NfL are age dependent, but values > 289 pg/ml are indicative of axonal damage of unknown specificity, whereas NfL values > 2.200 pg/ml can be found in patients with amyotrophic lateral sclerosis. NfL ratio is calculated by dividing CSF NfL by serum NfL. The increased NfL values in serum and CSF of all investigated patients reflect relevant axonal damage in affected individuals

*P* values show the comparison between responders and non-responders with a *t*-test and a standard *α* = 0.05. Due to the limited sample size (nine vs. three subjects), these should only be interpreted on an exploratory level. Responders: patients #1–9, non-responders: patients #10–12

↑ increased, *Ab* antibodies, *CASPR2* contactin-associated protein 2, *CSF* cerebrospinal fluid, *CT* computed tomography, *EEG* electroencephalography, *IgA%* intrathecal fraction IgA, *IgG%* intrathecal fraction IgG, *IgM%* intrathecal fraction IgM, *IIFT* indirect immunofluorescence technique, *IL-6* interleukin-6, *MRI* magnetic resonance imaging, *nd* not determined, *NfL* neurofilament light chain, *NMDAR* *N*-methyl-d-aspartate receptor, *PET–CT* Positron emission tomography–computed tomography, *SAH* subarachnoid hemorrhage

All patients received IVIg treatment at a median of 25 days (range 10–37) after acute encephalopathy onset and a median of 48 days (range 32–54) after hospital admission as *ultima ratio* therapy. Nine of 12 patients (#1–9, 75%, responders) presented milder symptoms at a median of 4 days (range 1–6) after IVIg initiation (Table 2). Three of 12 patients did not improve (#10–12, 25%, non-responders) and died of sepsis. 67% of responders had a negative CAM-ICU at discharge, while RASS improved from − 3 before therapy to 0 at discharge. Median GCS improved from 6 (range 3–12) to 14.5 (range 11–15), median GOS from 2 (range 2–3) to 3 (range 3–4) and median mRS from 5 (range 4–5) to 4 (range 3–5, Table 4). Before IVIg therapy, all patients required sedative (benzodiazepines, dexmedetomidine, clonidine, phenobarbital, propofol, esketamine, opiates) and/or neuroleptic or other CNS medication (risperidone, quetiapine, citalopram, amantadine) due to continuous encephalopathy, ongoing ventilation and ICU treatment. After IVIg administration, sedative or neuroleptic medication was successfully reduced in seven of 12 cases (Table 2). Patient #6 improved initially but died later due to sepsis. No adverse events attributable to IVIg therapy were observed.

**Table 4:** Results, primary clinical outcome

	Before IVIg	Min, Max	After IVIg	Min, Max	Discharge	Min, Max	
CAM-ICU	78% not possible		56% not possible		67% negative		Responder (*n* = 9)
	22% positive		44% positive		22% positive 11% N/A		
RASS	− 3	− 5, 2	0	− 4, 2	0	0, 1	
GCS	6	3, 12	11	5, 14	14.5	11, 15	
GOS	2	2, 3	3	2, 3	3	3, 4	
mRS	5	4, 5	5	4, 5	4	3, 5	
CAM-ICU	67% not possible		67% not possible				Nonresponder (*n* = 3)
	33% positive		33% negative		N/A		
RASS	−4	− 5, 2	− 3	− 4, 0	N/A		
GCS	9	3, 10	9	4, 11	N/A		
GOS	2	2, 3	2	2, 3	N/A		
mRS	4	3, 5	4	3, 5	N/A		

CAM-ICU results are “not possible” if RASS is -4 or -5. N/A is stated if patient died before discharge

*CAM-ICU* Confusion Assessment Method for Intensive Care Unit, *GCS* Glasgow Coma Scale, *GOS* Glasgow Outcome Scale, *IVIg* intravenous immunoglobulins, *mRS* modified Rankin Scale, *N/A* not applicable, *RASS* Richmond Agitation–Sedation Scale

Mean values of NfL in serum (responders 392 pg/ml, range 31–914 pg/ml), non-responders 2751 pg/ml, range 1348–4153 pg/ml) and CSF (responders 2406 pg/ml, range 981–6359 pg/ml, non-responders 14271 pg/ml, range 2215–28099 pg/ml) were higher in non-responders than in responders (Table 3). Similarly, mean values of IL-6 before IVIg treatment were lower in responders (98 ng/l, range 19–252 ng/l) compared to non-responders (438 ng/l, range 106–1087 ng/l) and decreased over the course of the treatment in responders (at discharge: 39 ng/l, range 7–99 ng/l) while increasing in non-responders (before death: 2983 ng/l, range 61–5905 ng/l, Table 3).

This case series demonstrates a therapeutic effect of IVIg in treatment-refractory COVID-19-associated acute encephalopathy. The pathophysiology of this entity remains speculative, but IVIg response points towards an immune-mediated mechanism, possibly succeeding COVID-19-induced cytokine release syndrome with BBB dysfunction and macrophage immigration or microglia activation, well in line with a neuroinflammatory hypothesis of COVID-19-associated acute encephalopathy [10]. The high frequency of intrathecal antineuronal antibodies detected by indirect immunofluorescence is in line with previous publications [8, 11] and might be a surrogate marker for autoimmune-mediated mechanisms, but whether they reflect specific or unspecific binding against CNS target epitopes causing acute encephalopathy currently remains open; especially since similar findings can be detected in asymptomatic persons. Immune-regulatory effects of IVIg are pleiotropic and involve Fc-receptor binding on macrophages and microglia, inflammation suppression including cytokines and chemokines, and transformation of activated microglia into a protective phenotype leading to reduction of neuronal cell death [12].

To date, one report described positive effects of IVIg on COVID-19-associated acute encephalopathy in five patients, but the therapeutic regimen overlapped with Tocilizumab and standardized methodology was lacking [13].

In our well-characterized cohort using standardized scores documenting responders and non-responders, natural remission coinciding the suspected IVIg effects cannot be excluded, but the long preceding interval without progress and the rapid improvement after IVIg initiation suggest a causal relationship. Due to the retrospective nature of our report and the individual treatment attempt as *ultima ratio* therapy, a control group without IVIg treatment is lacking. Unresponsiveness in three cases remains unexplained and might indicate variability in pathophysiological mechanisms of encephalopathy. Interestingly, IVIg non-responders presented very high NfL levels in serum and CSF that might also reflect a more intense neuronal damage leading to a more severe encephalopathy than in responders. Moreover, considering the fatal sepsis development in non-responders, the neurological improvement after IVIg therapy might have been masked by general disease severity with multi-organ failure. Of note, patient #1 started to improve within 1 day after beginning of IVIg treatment, which could be a coincidence. However, a more impressive and, thus, clearly definable improvement was seen 3–4 days after treatment of IVIg, which is why we tend to attribute the clinical change to our therapy.

In conclusion, IVIg are a promising immunotherapy for severe treatment-refractory COVID-19-associated acute encephalopathy. Further prospective controlled studies are required to validate safety and efficacy, monitor long-term outcome, and explore the mechanisms of the therapeutic IVIg effect.

## Data Availability

Anonymized data will be shared upon request from any qualified investigator.

## References

[1] Helms J, Kremer S, Merdji H, Schenck M, Severac F (2020). Delirium and encephalopathy in severe COVID-19: a cohort analysis of ICU patients. Crit Care.

[2] Slooter AJC, Otte WM, Devlin JW, Arora RC, Bleck TP (2020). Updated nomenclature of delirium and acute encephalopathy: statement of ten societies. Intensive Care Med.

[3] Cao A, Rohaut B, Le Guennec L, Saheb S, Marois C (2020). Severe COVID-19-related encephalitis can respond to immunotherapy. Brain.

[4] Pugin D, Vargas MI, Thieffry C, Schibler M, Grosgurin O (2020). COVID-19-related encephalopathy responsive to high-dose glucocorticoids. Neurology.

[5] Dogan L, Kaya D, Sarikaya T, Zengin R, Dincer A (2020). Plasmapheresis treatment in COVID-19–related autoimmune meningoencephalitis: case series. Brain Behav Immun.

[6] Perrin P, Collongues N, Baloglu S, Bedo D, Bassand X (2021). Cytokine release syndrome-associated encephalopathy in patients with COVID-19. Eur J Neurol.

[7] The RECOVERY Collaborative Group (2021). Dexamethasone in hospitalized patients with Covid-19. N Engl J Med.

[8] Franke C, Ferse C, Kreye J, Reincke SM, Sanchez-Sendin E (2021). High frequency of cerebrospinal fluid autoantibodies in COVID-19 patients with neurological symptoms. Brain Behav Immun.

[9] Jarius S, Pache F, Körtvelyessy P, Jelčić I, Stettner M (2022). Cerebrospinal fluid findings in COVID-19: a multicenter study of 150 lumbar punctures in 127 patients. J Neuroinflamm.

[10] Cerejeira J, Firmino H, Vaz-Serra A, Mukaetova-Ladinska EB (2010). The neuroinflammatory hypothesis of delirium. Acta Neuropathol.

[11] Song E, Bartley CM, Chow RD, Ngo TT, Jiang R (2021). Divergent and self-reactive immune responses in the CNS of COVID-19 patients with neurological symptoms. Cell Rep Med.

[12] Häußler V, Daehn T, Rissiek B, Roth V, Gerloff C (2020). Intravenous immunoglobulin (IVIg) induce a protective phenotype in microglia preventing neuronal cell death in ischaemic stroke. NeuroMol Med.

[13] Muccioli L, Pensato U, Bernabè G, Ferri L, Tappatà M (2020). Intravenous immunoglobulin therapy in COVID-19-related encephalopathy. J Neurol.

